# Editorial: Women in molecular and cellular oncology

**DOI:** 10.3389/fonc.2023.1257780

**Published:** 2023-09-04

**Authors:** Petranel T. Ferrao, Laura Rosanò, Valeria Poli, Shilpa S. Dhar, Ana Paula Lepique

**Affiliations:** ^1^Independent Researcher, Adelaide, SA, Australia; ^2^Precision Medicine, South Australian Health and Medical Research Institute, North Terrace, Adelaide, SA, Australia; ^3^Corpallium Pty Ltd, Adelaide, SA, Australia; ^4^Plena Vitae Therapies Pty Ltd, Adelaide, SA, Australia; ^5^Institute of Molecular Biology and Pathology, Consiglio Nazionale delle Ricerche (CNR), Rome, Italy; ^6^Department of Molecular Biotechnology and Health Sciences, University of Turin, Torino, Italy; ^7^Department of Molecular & Cellular Oncology, The University of Texas M. D. Anderson Cancer Center, Houston, TX, United States; ^8^Department of Immunology, Instituto de Ciências Biomédicas, Universidade de São Paulo, São Paulo, SP, Brazil

**Keywords:** gender equality, STEMM, cancer, oncology clinical trials, treatment cross-resistance, female leadership, diversity

Despite clear evidence that diversity increases the quality and impact of science (1–3), there remains more to be done to achieve gender equality. While the number of female students and post-doctoral fellows can equal or even exceed that of males in most organizations, the ratios are reversed at more senior levels with a much greater representation of scientists identifying as male (4). Male scientists are also more likely to be cited in papers and to successfully secure research grants (5, 6). In relation to awards and recognition, less than 4% of Nobel Prizes for science have been awarded to women (7).

Various organizations and associations are endeavoring to bridge this gap by implementing new approaches that support women, such as achieving equitable conditions of work, recruitment and promotion, appraisal, training, and pay without discrimination. This inaugural Research Topic “*Women in molecular and cellular oncology*” is one of such initiatives providing a platform that promotes STEMM research by women, by inviting first or senior author contributions from scientists identifying as female.

In this Research Topic, there are 14 articles led by women on various aspects of several cancer types. Waldhorn et al. have compiled data from clinical trials from the last two decades, highlighting women underrepresentation in leading positions in oncology clinical trials. Although the percentage of female principal investigators in oncology clinical trials has been slowly increasing, mainly with respect to cancers more common in females, such as breast cancer, the increase is slow worldwide and more so in Asia.

## Breast cancer (BC)

Cholesterol metabolism is gaining increasing attention in BC, although its role remains controversial. Centonze et al. discussed new understanding of cholesterol homeostasis and summarized key findings of recent preclinical and clinical studies investigating cholesterol metabolism and its derivatives. They provided discussion on the therapeutic effects of natural compounds and cholesterol-lowering drugs in BC treatment, opening a window for new innovative combinatorial therapies, although future work will be needed to evaluate their effective therapeutic potential.

Wang et al. examined the predictive power of breast cancer staging based on positive lymph node ratio (LNR), demonstrating that patients with apical or infraclavicular/ipsilateral supraclavicular lymph node metastasis (APN(+)) had a significantly worse prognosis than APN(−) patients in the same LNR staging group. Accordingly, exclusion of APN(+) patients from the LNR classification significantly improved its predictive power. This study contributes to improving the precision of LNR classification for APN (–) patients.

## Cervical and endometrial cancer

Wen et al. reported that the prevalent genomic mutations in Chinese cervical cancer patients were not significantly different when compared to TCGA data of patients from western countries. In both groups, DNA damage repair (DDR) gene alterations were significantly correlated with hypoxia features and increased Tumor Mutational Burden, but not with immunosuppression as previously proposed. The authors therefore suggest that DDR alterations may not be robust predictors of Immune Checkpoint Inhibitor responsiveness in cervical cancer.

Mahajan et al. explored the changes in the expression of TET enzymes and steroid hormone receptors in response to hormones in endometrial cancer cells. Their results suggest that TET gene expression and protein levels are cell-specific and imply possible co-regulation of the expression of steroids and steroid receptors, prompting future studies on how these expression patterns could regulate endometrial biology and interrelate in endometrial cancers.

## Perivascular epithelioid cell tumors (PEComas)

PEComas are rare and mostly benign soft tissue neoplasms, only rarely presenting as malignant with poor prognosis, in part due to resistance to conventional chemotherapy. Sui et al. described a patient with chemotherapy resistant metastatic uterine PEComa displaying a partial response to combined treatment with the mTOR and VEGR inhibitors Everolimus and Apatininb. Treatment was chosen after targeted next-generation sequencing, corroborating work by others supporting target-specific therapy for malignant PEComas.

Butz et al. reported a novel TP53 germline splice mutation in a metastatic PEComa and a sinonasal carcinoma. This discovery contributes to the growing number of newly identified germline TP53 variants identified through Next Generation Sequencing, which expands the understanding of Li-Fraumeni syndrome and its association with a wider range of cancer predispositions. The study demonstrated locus-specific loss of heterozygosity in the PEComa, suggesting that the splicing mutation plays a causal role in its development. This study represents the first evidence linking an abnormal TP53 mutation to PEComa.

## Lung cancers

Mixed small cell lung cancer (SCLC) and large cell neuroendocrine lung carcinomas (LCNEC) are rare and poorly characterized tumors. Zhu et al. described a tumor containing 35% LCNEC and 65% SCLC, suggesting a common clonal origin with dual mutations in TP53 and RB1. This is an important contribution towards the understanding of this type of cancer, characterized by high genomic stability and with few therapeutic options.

ROS1 rearrangements occur in 1-2% of non-small cell lung cancer (NSCLC) cases, with about 10 fusion partners identified so far. Wei et al. reported a case where a stage IV NSCLC patient harboring a novel TPR-ROS1 fusion showed a rapid but transient response to Crizotinib but resistance to Ceritinib, with a pulmonary nodule negative for PD-L1 staining but displaying the TPR-ROS1 fusion. After the transient Crizotinib response, the patient responded well to chemotherapy. This case highlights TPR-ROS1 as an oncogenic driver, encouraging further research to understand resistance mechanisms and develop effective treatments.

## Retinoblastoma

Ke et al., by combining simplified RNAseq data with functional studies in a human retinoblastoma cell line, hypothesize that the downregulation of miR-211-5p is associated with the upregulation of GDNF and of a metabolic pathway leading to carboplatin excretion and drug resistance.

## Hematological malignancies

Scripicca et al. described the impact of cyclin-dependent kinases (CDK) inhibitors (CKI) on cancer progression, providing a systematic overview of the key alterations of INK4 or CIP/KIP family members and their function in hematological malignancies. They noted the need for development of novel CDK inhibitors with reduced side effects for cancer treatment.

Resistance to BCR-ABL Tyrosine Kinase Inhibitors, a game changer treatment in Chronic Myeloid Leukemia (CML), is mainly, but not always, due to mutations in BCR-ABL. Elias et al. systematically reviewed the literature on CML focusing on differential expression of miRNAs, bioinformatically identifying their main target genes and associated pathways linked to resistance, which included genomic instability, proliferation, apoptosis, differentiation, and migration.

Chronic Lymphocytic Leukemia (CLL) is a common lymphoid malignancy linked to dysregulated expression of anti-apoptotic and pro-apoptotic members of the Bcl-2 family. Boncompagni et al. demonstrated that glycerophosphoinositol (GroPIns) can induce expression and activity of the pro-apoptotic family member Bax *via* both binding and modulating SHP-1 and directly interacting with Bax to promote its activation and recruitment to the mitochondria. These data suggest that GroPIns treatment may help overcoming the apoptosis defect of CLL cells, enhancing the effects of other drugs including the Bcl-2 inhibitors.

## An emerging common issue across multiple cancers

Treatment resistance is a common problem in cancer therapy as mentioned in some of the articles outlined above. With the recent approval of many new therapeutics, it is common for patients to receive a variety of different treatments throughout their cancer journey and acquire cross-resistance. Discussing the current literature on drug resistance and focusing on cross-resistance to sequential therapeutics and the underlying molecular mechanisms in diverse tumor types, Loria et al. suggest that real-world patient data is often more complex than predicted from clinical trials and offer perspectives for the development of more effective personalized treatment strategies.

There is still much more to be done in the field of molecular and cellular oncology to improve our understanding of the underlying biological characteristics of cancers, particularly in relation to treatments. As we face some major challenges, revealed by ‘real world’ observations of cross-resistance to sequential treatments, there is an on-going need to drive meaningful progress.

Since diversity in scientific teams enhances creativity and innovation (8), and increases the quality and impact of science, having input and contribution from a broad spectrum of researchers, irrespective of gender or other differences, to develop, produce and present quality research is an advantage. Accordingly, it is essential to implement strategies that actively support underrepresented groups, particularly women that would like to pursue roles in the STEMM field now and in the future.

Waldhorn et al. discussed the necessity of affirmative action to increase female leadership representation in medicine and science in general. As a group of women who have co-edited this inaugural 1st edition of *Women in molecular and cancer oncology 2021*, we feel strongly about supporting the need for more action to gain gender equality at all levels and across all sectors that contribute to the advancement of the oncology field. As we strive to build more opportunities, such as this topic that highlights and showcases the research advances being led and driven by women in the field, we believe that by supporting the path to gender equality, the quality and impact of research will also improve, providing benefits for all cancer patients.

## Author contributions

PF: Conceptualization, Project Administration, Writing – original draft, Writing – review & editing. LR: Writing – original draft, Writing – review & editing. VP: Writing – original draft, Writing – review & editing. SD: Writing – original draft, Writing – review & editing. AL: Writing – original draft, Writing – review & editing.
